# Investigation of Toxic Metals in the Tobacco of Different Iranian Cigarette Brands and Related Health Issues

**Published:** 2012

**Authors:** Alireza Pourkhabbaz, Hamidreza Pourkhabbaz

**Affiliations:** 1 *Department of Environmental Science, Agriculture Faculty, Birjand University,Birjand, Iran*; 2 *Department of Environmental Science, Natural Resources Faculty, Behbahan University, Behbahan, Iran*

**Keywords:** Cigarette tobacco, Health, Smoking, Toxic Metal

## Abstract

**Objective(s):**

The primary objective of this study was to determine whether local and imported cigarette brands used in , have elevated levels of metals or not. The produced data of cigarette brands are compared both with each other and with the existing brands in different countries.

**Materials and Methods:**

In present study, nineteen various cigarettes brands were randomly purchased from the commercially available cigarettes in Iranian market (Birjand city) including local Iranian branded and imported cigarettes. All samples were analyzed for heavy metals, viz. Cd, ,Cu ,Co Ni, Zn and Pb by graphite furnace atomic absorption spectrophotometer after microwave-assisted wet digestion method with nitric and perchloric acids.

**Results:**

The observed average metals concentrations for cadmium in all cigarette brands was 2.71 and ranging 1.76 to 3.20, copper 9.7 (5.18-17.6), cobalt 4.42 with range of 2.57-6.49, nickel 17.93 (10.0-30), zinc 27.02 (18.1-42.2) and value for lead was 2.07 with range of 1.05 to 3.10 (µg/g dry weight) and mean metals content per cigarette was also measured. The produced data of imported and local cigarette brands are discussed and compared together and with studies from elsewhere.

**Conclusion:**

The investigation may confirm that the level of metal contents in Iranian cigarettes is similar to the other parts of the world. However, the concentration of these metals was slightly higher in comparison with other investigation.

## Introduction

Some of the trace metals in biological samples derive from their essentiality, as well as from their potentially toxic effects in living organisms. That is, pollution due to heavy metals has acquired importance, since these metals are highly toxic for humans and for the whole ecology. 

Cobalt, nickel, copper, zinc, lead and cadmium, are metals with known toxic properties (1, 2). Cobalt is an essential trace element for human since it is a necessary constitute for the formation of vitamin B_12_ (3, 4). However, in higher concentrations, Co is toxic to humans and it is also toxic to the cells as it inhibits cellular respiration and enzymes of citric acid cycle (5).

Nickel is usually present in high concentrations in the liquid wastes which are released directly into the environment without any pre-treatment (6). It is one of the stable and persistent environmental contaminants since it cannot be biologically or chemically degraded or destroyed unlike many other organic toxic pollutants. Therefore, the metal has become a serious worldwide environmental problem. Although nickel is a trace element required for living organisms, it is toxic when ingested in large amounts. Epidemiologic and experimental studies of nickel related cancer evaluated and concluded that nickel compounds are also well recognized as carcinogens (7).

As trace elements, copper and zinc also possess an underlying role for the function of different enzymes and other cellular proteins, however, too much intracellular accumulation may culminate in toxicity. In case of excessive intracellular accumulation, Cu becomes toxic, and it plays a role in initiating the generation of detoxification of reactive oxygen species and apoptotic processes. (8). Zinc becomes toxic in the case of excessive intake playing a role in induction of pathological conditions that have been associated with oxidative stress (9).

The environmentalist and government health organization are much more worried about high presence lead and cadmium as non-essential. These metals are potentially hazardous and carcinogenic even in small concentration. They also are a toxic trace metal that has no biological function in humans and plants but also has an accumulative metabolic poison (bio-accumulate) with physiological and neurological effects (10-12). According to Harrison and Laxen, (13) and WHO, (14,15) lead and cadmium are highly toxic metals and are capable of causing serious effects on the brain, kidneys, nervous system, intelligence quotient (16) and red blood cells. In these organs cadmium is bound to a small protein called metallothionin (17). Anyhow, acceptable concentrations of Pb and Cd in human blood are below 100 and 10 μg/l, respectively, depending on the subject’s age and gender (18). 

There are a number of inorganic toxic elements and essential element present in tobacco along with organic carcinogens. Tobacco plant is open to absorb and accumulate heavy metal species from the soil into its leaves (19) or deposited on tobacco leaves from air (20). Tobacco is a sensitive plant prone to many diseases.

Elinder *et al* in 1983 (21) and Galazyn-Sidorezuk *et al* in 2008 (22), too, reported that tobacco plant easily take up metals from soil and concentrated them in leaves. This contamination is different in each country in which the tobacco plant is harvested and processed. Thus tobacco and cigarettes can generally accumulate metals such as Pb, Cd, Zn, Ni and Cu preferentially (23-25). More studies by Ebisike *et al* (26), Barlas *et al* (27), Yang *et al* (28), Verma *et al* (19) and Schneider and Krivan (29) showed high levels of toxic elements in cigarettes tobacco. 

Heavy metals, through inhalation of smoking, easily get incorporated into users’ bodies during smoking. Numerous previous data gives evidence that metals exist in higher concentration in tissues of smokers than in non-smoking persons (19, 22). Thus, cigarette tobacco is very harmful, toxic and genotoxic for human health (30). Therefore, the concentration of heavy metals in cigarette brands is of importance because of its toxicological effects (31). 

In , cigarettes are used liberally for smoking or for pleasure. Many young or adult men and women enjoy smoking worldwide; therefore the monitoring of heavy metals in tobacco is essential for protection of the environment and of our health. In , whether nobody made data or it is inaccessible yet, hence, heavy metal is very dangerous for health on smokers and potential smokers. Thus, in this investigation, 76 samples of different types of cigarettes belonging to various brands (local and imported cigarettes) were collected to make fresh data and measure six heavy metals (lead, nickel, cobalt, copper, zinc and cadmium). The produced data of imported and local cigarettes are discussed and compared with each other and with the existing ones in different countries.

## Materials and Methods

***Instrumentation and reagents***

Varian spectra 800 were coupled with GTA 100 electro thermal atomizer unit, graphite furnace atomic absorption spectrometer was used to determine the concentrations of metals. The temperature program and recommended parameters for trace elements at this instrument technique have been shown in Table 1. 

A Sartorius analytical balance (Model A 120 S, ), Filter Paper Circles 110 mm diameter (ashless-Germany), PTFE (Polytetrafluoroethylene) vessel, microwave (VT 6130 M, Heraeus instruments, ) were employed throughout the procedure. 

Agate ball mixer mill (MM-2000 Haan, ) such as Wiley mill, was used for grinding the cigarette tobacco. Sieves made of nylon with mesh sizes of Ø <65 were used to study the influence of particle size on extraction.

All chemical material was used of Merck company. The Teflon boxes and plastic boxes were washed with 200 ml acid acetic 65% plus 5000 ml bidistilled water, and volumetric flasks with 50 ml acid acetic 65% plus 5000 ml bidistilled water, then they were washed two times with distilled water to remove any contamination. 

***Preparation of tobacco samples***

Nineteen different brands of commonly sold cigarette in Iran market were randomly purchased from both imported (n= 11) and locally manufactured cigarettes (n= 8) (Table 3). 

Four packs of different batch number (for purpose of random sampling) and five of each composite batch of all branded cigarettes under study was used. For analysis of trace elements in cigarette tobacco, we separated all components of cigarette, tobacco, filter and wrapping paper of cigarettes. The average weight of each cigarette was determined by weighing 5 sticks of each brand whose means was about 1.02 g, and 82 mm in length.

As soon as practicable after collecting, tobacco samples are commonly dried in a beaker, then grounded in an agate grinder (300 rpm) and sieved to obtain a < 65 µm fraction.

***Conventional acid digestion method***

The method of acid digestion of solid samples is a total digestion technique for most samples. Digestion with acid or acid mixtures has long been the traditional method for the determination of metals in plants**.** It is very strong acid digestion that will dissolve almost all elements that could become environmentally available.

**Table 1:** Temperature program and recommended parameters for GFAAS technique

Element	Drying (°C)	Melting point (°C)	Boiling point (°C)	Wavelength (nm)	Detection limit (ng/ ml)
Pb	130	850	1800	283.3	7.9
Ni	130	1000	2400	232.0	2.3
Cd	130	800	1600	228.2	0.22
Co Cu	130 130	950 800	2200 2300	240.7 324,7	- 1.4

For the determination of metals, 1 g of samples (dry weight, DW) was placed in a PTFE vessel and allowed to digest with a mixture of acidified with 10 ml of HNO_3_ (65%) and HCl (37%) with a ratio of 4:1 (v/v) by heating them in microwave (wet-ashed) under high pressure (200 Atm) by 180 °C for 12 hr as described by Feldmann (32). PTFE bomb was kept for an hour to cool and was carefully opened. After cooling, 10 ml of de-ionized water was added. 

***Analysis of standard reference materials***

Validation of the trace element measurements n tobaccos was performed in laboratory by analysis of standard reference materials to confirm the method used for the analysis of heavy metals. 

Reference standards for tobacco are few. In this investigation, in order to control the accuracy and the reproducibility of the chemical measurement process has been evaluated by analyzing certified samples of Pine Needles and Orchard Leaves from the United States National Bureau of Standards.

All samples were analyzed for heavy metals, viz. Cd, Cu, Co Ni, Zn and Pb by Graphite furnace atomic absorption spectrophotometer (Varian spectra 800) in µg/g dry weight (DW).

***Statistics***

The original data were processed by one way ANOVA analysis and Microsoft Excel software to evaluate statistical model. Student’s t-test was used for the statistical analysis of the differences in heavy metals between imported and local cigarettes samples. The statistical evaluation of factors investigated was carried out using the Statistical Package for Social Sciences (SPSS, Version 16). 

## Results

The results of reference standards for tobacco were in good agreement with the certified values for trace metals and are shown in Table 2. This procedure was repeatedly performed after every five readings.

The precision of the method was studied on two different types of cigarettes, local and imported cigarettes sold in . The metals including lead, nickel, copper, cobalt, zinc and cadmium contents of several cigarette tobacco products are listed in Table 3. The standard deviations, which are indicated in the same table for trace elements, mostly reflect the variability of metal levels in the samples and in different batches of the cigarettes.

Local cigarettes had a mean cadmium concentration 3.12 µg/g (DW) with a standard deviation of 0.06 whereas imported brands showed a cadmium concentration average 2.47 µg/g (DW) with a standard deviation of 0.41 µg/g. These results were followed by lead, copper, cobalt, zinc and nickel 2.27±0.27, 13.04±2.99, 4.58±1.2, 29.4±7.7 and 22.41±5.4 µg/g for local brands and 1.69±0.44, 7.8±2.3, 4.33±1.3, 25.6±5.03 and 15.32±4.7 µg/g for imported cigarettes respectively. Therefore, higher concentration of cadmium, nickel, copper and lead were observed in local cigarette brands as compared to imported cigarette brands. On other hand, among cigarette brands, the highest concentration of cadmium element were found in Sima (3, 2 µg/g), (3, 19 µg/g), Mond (3, 10 µg/g) (imported cigaretts) and Bahman with 3, 05 µg/g (local brand), whereas lowest cadmium content was in Lavan and cigarettes with 1, 76 and 1, 95 µg/g, respectively.

**Table 2:** Results obtained for the standard reference materials together with certified value (µg g^-1^) (n=3)

Element	Found SD	NIST 1571 SD	Found SD	NIST 1575 SD
Cd	0.1 0.1	0.11 0.0	0.220.2	0.230.1
Ni	1.1 0.5	1.5 0.3	1.480.4	1.470.3
Cu	11 0.3	11.8 0.6	2.20.5	2.80.3
Pb	43 4.2	44 2.0	0.140.1	0.170.1
Zn	22 7.9	25 2.0	36.53.1	382.2
Co	0.30.2	0.20.1	0.040.0	0.060.0

SD= Standard deviation

**Table 3:** Mean concentration of different metals along with their standard deviation (SD) in different brands of cigarette tobacco (µg/g)

Brands	Cd	Cu	Co	Zn	Ni	Pb
	Mean SD	Mean SD	Mean SD	Mean SD	Mean SD	Mean SD
	2.77 1.0	5.18 1.0	4.48 1.2	23.55 2.7	15.9 3.2	1.05 0.1
Magno	2.14 0.5	12.86 1.1	3.95 1.4	25.13 2.0	21.2 4.2	1.10 0.2
Tir	2.15 1.0	8.70 0.7	6.08 1.9	42.16 5.4	14.48 2.1	2.00 0.9
	1.95 0.2	8.20 0.8	5.26 2.0	31.44 2.8	12.45 3.2	1.80 0.3
Ordibehesht	2.51 0.3	13.51 1.4	4.67 1.8	20.23 1.9	21.98 7.2	1.90 0.1
Winston	2.42 0.3	6.61 0.7	6.23 2.1	31.77 3.3	15.63 6.4	1.70 0.3
Farvardin	3.10 1.1	10.40 1.1	4.17 0.8	27.75 2.5	20.61 5.1	2.50 0.9
Lavan	1.76 0.1	15.2 1.7	3.40 0.7	35.74 5.0	21.93 8.7	2.10 0.9
Pine	3.00 1.0	6.02 0.6	3.33 0.8	21.33 2.2	18.71 5.6	1.20 0.1
Esse	2.50 0.9	8.51 0.9	3.31 0.8	23.10 2.8	11.52 1.9	1.40 0.1
	2.76 1.0	14.02 1.5	2.8 0.7	32.57 3.1	30.01 9.8	1.60 0.1
Pleasure	2.61 0.9	6.34 0.7	3.68 0.9	25.59 2.7	16.75 4.5	1.90 0.1
Mond	3.14 0.9	11.53 1.2	2.65 0.9	18.12 1.7	10.03 2.4	2.35 0.8
Day	3.06 1.0	11.90 2.2	5.68 1.3	24.63 2.9	19.02 6.1	3.10 0.9
Marlboro	3.08 1.0	8.34 0.7	2.57 0.8	20.57 2.2	11.81 1.8	2.85 1.0
	3.19 0.9	6.62 0.7	5.22 1.0	31.78 3.4	12.73 2.1	2.40 0.9
Three stars	3.10 0.9	8.73 0.7	6.49 1.2	22.34 2.5	26.08 8.5	2.90 0.7
Sima	3.20 0.9	5.50 0.6	4.83 0.9	32.51 3.1	10.95 1.6	2.65 0.7
Bahman	3.05 1.0	17.59 1.8	5.27 1.0	23.20 2.7	28.89 9.5	2.80 0.9
Average	2.71	9.7	4.42	27.02	17.93	2.07
Min	1.76	5.18	2.57	18.1	10.03	1.05
Max	3.20	17.6	6.49	42.2	30.00	3.10

**Table 4:** Mean concentration with standard deviation (SD) of different metals per Iranian cigarette (µg)

Brands	Cd	Cu	Co	Zn	Ni	Pb
	Mean SD	Mean SD	Mean SD	Mean SD	Mean SD	Mean SD
	1.04 0.08	1.04 0.21	0.91 0.12	5.01 1.01	3.17 0.91	0.21 0.02
Magno	1.02 0.04	2.18 0.80	0.77 0.07	3.10 1.01	4.15 1.02	0.32 0.04
Tir	0.92 0.07	1.62 0.42	1.25 0.41	9.21 2.01	2.97 0.91	0.90 0.02
	0.54 0.10	1.57 0.63	1.06 0.13	6.32 2.10	2.51 0.85	0.41 0.01
Ordibehsht	1.00 0.10	2.70 0.11	0.94 0.14	4.14 1.01	4.44 1.21	0.98 0.10
Winston	1.01 0.05	1.42 0.41	1.33 0.85	7.02 2.02	3.33 1.05	1.01 0.09
Farvardin	1.04 0.08	2.11 0.61	0.84 0.06	4.07 1.01	4.16 1.24	1.25 0.02
Lavan	0.85 0.02	3.04 0.91	0.69 0.23	3.10 1.02	4.46 1.34	0.54 0.10
Pine	0.98 0.10	1.37 0.58	0.77 0.11	5.14 1.10	4.34 1.23	0.95 0.12
Esse	0.97 0.04	1.76 0.57	0.71 0.18	5.06 1.01	2.46 0.75	0.93 0.09
	0.57 0.02	2.59 0.88	0.54 0.09	2.02 0.71	5.76 1.85	0.75 0.10
Pleasure	0.99 0.04	1.30 0.31	0.75 0.23	5.21 1.03	3.41 1.08	0.84 0.10
Mond	1.10 0.08	2.21 0.82	0.52 0.10	4.18 1.01	1.95 0.24	0.89 0.09
Day	1.32 0.56	2.5 0.7	1.2 0.4	5.27 1.61	2.85 1.03	1.12 0.10
Marlboro	1.24 0.43	1.7 0.35	0.55 0.2	4.17 0.98	2.51 1.12	1.21 0.12
	1.12 0.21	1.2 0.2	1.02 0.8	6.26 1.70	2.48 1.02	0.85 0.05
Three stars	1.08 0.12	1.7 0.2	1.33 0.8	5.21 1.03	5.35 1.86	1.21 0.09
Sima	1.34 0.13	1.0 0.1	0.94 0.2	6.34 1.83	2.12 1.03	1.35 0.11
Bahman	1.25 0.11	3.4 1.1	1.02 0.4	3.06 0.75	5.59 1.28	1.21 0.05
Average	1.02	1.9	0.9	5	3.58	0.89
Min	0.54	1.0	0.52	2.0	1.95	0.21
Max	1.34	3.4	1.33	9.2	5.76	1.35

The content of trace elements per cigarette was also calculated based on weighing each cigarette (Table 4). It is so important to point out that the cadmium level of local brands (1.21±0.11 µg per cigarette), which is within the range of 1.08-1.34 µg used in this study was slightly higher than values for imported brands (0.91±0.18 µg per cigarette) ranging from 0.54 to 1.04 µg, but no significant different was found respectively. This fact was for lead in Iranian cigarettes 1.12±0.18 (0.85-1.35) and 0.76±0.32 (0.21-1.25) µg per cigarette, respectively (Table 4). The concentration of other elements including; nickel, cobalt, zinc and copper element per cigarette is also indicated in Table 4.

## Discussion

The concentration of metals in both cigarette brands group follows almost the same trend; 

Zn > Ni > Cu > Co > Cd> Pb. 

Concentration between imported and local cigarettes is statistically significant at 95% confidence level. Of course, there was relatively a wide range of variation in mean concentration of elements in imported brands (for example, coefficient of variation of 16.67 % for Cd and 25.96% for Pb ) in comparison with local brands (coefficient of variation of 1.98% for cadmium and 10.14% for lead). Such inner brand variations in trace element concentration of tobacco products have been observed by others as well (19, 33). However, these variations could possibly be related to agriculture soil contents of trace metals on which tobacco leaves were cultivated (16, 34), farming fields close to roads and residential areas (30), the chemistry of tobacco leaves and finally to its processing (19). Nnorom *et al* (31) also reported, the mean metal contents of cigarettes varied markedly depending on the geographical area of production. However, it has not been possible to get any evidence to explain whether differences are related to the area of production or the extent of industrial development of the area.

Table 3 also showed the concentration range for cadmium in the tobacco in all Iranian cigarette brands from 1.76 to 3.20 µg/g with an average of 2.71±0.46 µg/g. This metal content was higher than its degree in (36), (35), (11) and some reported investigation on Table 5, but comparable with cigarette tobaccos of Yugoslavian (38), (37), and (21). Mussalo-Rauhama *et al* (39) investigated in different cigarettes and found the cadmium content ranging between 0.8 to 3.4 μg/g (DW). Compared with the reported results for Cd in the United Kingdom (0.90 μg/g) and Korean cigarettes (1.02 µg/g), the average Cd contents in Iranian cigarettes are 3.01 and 2.65 times higher than that of United Kingdom and Korea, respectively (33).

The mean Pb concentration in tobacco samples 2.07 µg/g, have significantly lower concentration than Indian cigarette (2.4 to 4.3 µg/g) (31, 35), UK 4.1 Pb µg/g (34), Ethiopia 6.07 Pb µg/g (37) and Pakistan with range 8.7 to 14.4 Pb µg/g (9) but almost agree with Andrade *et al* (11), Massadeh *et al* (24), Watanabe *et al* (23) and more published data on Table 5.

Zinc and copper are essential elements in plant function with low concentrations and aid in plant metabolism photosynthesis and activator of several enzymes. High concentrations of these metals are toxic in both plant and human (12). Zinc, nickel and copper have been found in all of the major cigarette components. Mean contents of these elements show that nickel significantly indicate higher concentration compared to existing published data in Table 5, whereas, zinc is comparable with Indian cigarette and significantly higher to Ohaio, Germany and India. Of course, copper contents in Iranian cigarettes are comparable with some reports in elsewhere (Table 5). Generally, estimated ranges of daily intakes of total Zn, Cu, Co, Pb and Cd: 8.8-14.4 mg, 10-30 µg, 50 µg; 8-10 µg and 0.4-1.2 µg, respectively (40, 41). 

Also, reported investigations on element concentration per cigarette are not much more, specially for nickel, cobalt, lead, copper and zinc. However, Iranian cigarettes show significant lower metal contents (1.02 Cd and 0.89 Pb µg per cigarett) in comparison with studies in Japan (42), Ethiopia (37) and Germany (43) and are almost comparable with studies done in China, France (23) and Poland (22). As mentioned early, this difference is because heavy metal concentrations vary in soil and air, it is expected that the results for heavy metal concentrations in tobaccos be also different.

**Table 5:** Comparison of the result of studies of trace elements contents of cigarettes in various countries and results of the present study

Trace metals						References
Cu	Cu	Zn	Co	Ni	Pb	
2.71	9.7	27.02	4.42	17.93	2.07	This study
0.90	13	31.9			0.74	(33)
1.02	7.73	38.5			1.35	(33)
		51.4				Ohaio (2)
0.4	18	29	0.91	3.6	1.6	(46)
0.18	4.13			2.23	0.64	(33)
1.7	2.45			0.22	1.02	(27)
1.95	9.7	49.8		2.4	1.2	(29)
2.64	12.9	55.62			2.67	(24)
0.5	7.89	8.57			14.53	(9)
0.9	39	39.5		3	4.3	(40)
0.45	14	27		8.79	1.94	(19)
2.48	12.70	36.22			6.07	(37)

It has been published in different studies (2, 21, 42, 44) that an average of 5- 30% of Cd and Pb respectively from one cigarette passed to mainstream smoke. 40-60% of cadmium inhaled via smoking (19) can directly enter the blood stream of the smoker easily (34), smoking more than 20 cigarettes daily can increase Cd contents in body by 10 folds (19 and references therein) and damage the body organs. On the other hand, pervious reports (12 and references therein) proved that excess zinc can prevent cadmium toxicology. That is, the ratio of zinc-cadmium is very important because cadmium toxicity cause greater activity in tissue function with zinc deficiency, thus cadmium may displace zinc from binding sites like enzymatic and organ functions. Therefore, the competition between Cd and Zn is evidenced by the fact that extra Zn can prevent Cd toxicity.

Other metals also accumulate in the blood stream by smoking (45). For example, WHO (15) and Galażyn-Sidorczuk *et al* (22) estimates 2–6% and 10% respectively, of Pb in cigarettes is inhaled by the smoker. Thus, the use of cigarette products not only damage the smokers but also effects on health of non smokers especially children, pregnant women and adults that result in high metals content in blood stream. 

Another concern is that other heavy metals such as Ni, Co and Cu may also be entering the lungs and bloodstream, and the effects may be additional or even synergistic with Cd, but experimental transfer coefficients between tobacco and smoke are not presently well-characterized for many heavy metals. If any of these low-dose effects proves to cause significant harm, then the role of enhanced levels of Cd and other heavy metals in cigarette smoke becomes rather more significant than currently appreciated (34).

## Conclusion

Since there was no sufficient data about heavy metals contents in Iranian cigarette brands, this study could provide a new data to be useful for health organizations in . Measured levels of toxic metals in cigarette tobacco brands show further information from a public health places. These results indicated that tobacco plant is a main source of many trace metals pollutants particularly cadmium, copper, zinc, nickel, and cobalt, which are uptaken from soil and then transferred to upper parts of plant or deposit on tobacco leaves via air. The investigation may confirm that the levels of metals contents in Iranian cigarettes is similar to other parts of the world, but the concentration of these metals was slightly higher in comparison with other investigation (Table 5) which could be related to soil characteristics and fertilizer practices. So it can extremely be increased by the effects of inhalation metals especially cadmium in places where smokers are present and it results in health problem more than what was assumed. 

Thus, these data suggest smokers in Iran could receive significantly higher exposures to various toxic and carcinogenic metals from cigarettes and have higher intake of trace metals particularly Cd and Pb (average 10%) than the non-smokers.
